# Supplementary material for: Pyruvate Dehydrogenase Kinase 1 inhibition mediated oxidative phosphorylation enhancement in cartilage promotes osteoarthritis progression
Source: BMC Musculoskelet Disord. 2023 Jul 20;24:597. doi: 10.1186/s12891-023-06585-6 (PMC10357736; doi:10.1186/s12891-023-06585-6)
Supplement: Supplementary file 1 — Additional file 1. [file 12891_2023_6585_MOESM1_ESM.zip › MF.Gsea.1653627316242/GOMF_CORE_PROMOTER_SEQUENCE_SPECIFIC_DNA_BINDING.html]

Details for gene set GOMF\_CORE\_PROMOTER\_SEQUENCE\_SPECIFIC\_DNA\_BINDING[GSEA]

|  || Dataset | 55457\_collapsed\_to\_symbols.cls.cls #OA\_versus\_normal.cls.cls #OA\_versus\_normal\_repos |
| Phenotype | cls.cls#OA\_versus\_normal\_repos |
| Upregulated in class | normal |
| GeneSet | GOMF\_CORE\_PROMOTER\_SEQUENCE\_SPECIFIC\_DNA\_BINDING |
| Enrichment Score (ES) | -0.42652544 |
| Normalized Enrichment Score (NES) | -1.5925182 |
| Nominal p-value | 0.01188455 |
| FDR q-value | 0.15576275 |
| FWER p-Value | 0.998 |
Table: GSEA Results Summary

  

Fig 1: Enrichment plot: GOMF\_CORE\_PROMOTER\_SEQUENCE\_SPECIFIC\_DNA\_BINDING      
 Profile of the Running ES Score & Positions of GeneSet Members on the Rank Ordered List

  

| SYMBOL | TITLE | RANK IN GENE LIST | RANK METRIC SCORE | RUNNING ES | CORE ENRICHMENT || 1 | PAX6 | paired box 6 [Source:HGNC Symbol;Acc:HGNC:8620] | 304 | 0.746 | 0.0196 | No |
| 2 | NR3C1 | nuclear receptor subfamily 3 group C member 1 [Source:HGNC Symbol;Acc:HGNC:7978] | 674 | 0.599 | 0.0255 | No |
| 3 | GTF2B | general transcription factor IIB [Source:HGNC Symbol;Acc:HGNC:4648] | 1315 | 0.455 | 0.0021 | No |
| 4 | PARK7 | Parkinsonism associated deglycase [Source:HGNC Symbol;Acc:HGNC:16369] | 1406 | 0.442 | 0.0207 | No |
| 5 | GBX2 | gastrulation brain homeobox 2 [Source:HGNC Symbol;Acc:HGNC:4186] | 1861 | 0.378 | 0.0073 | No |
| 6 | SNAPC3 | small nuclear RNA activating complex polypeptide 3 [Source:HGNC Symbol;Acc:HGNC:11136] | 1871 | 0.376 | 0.0284 | No |
| 7 | GTF2A1 | general transcription factor IIA subunit 1 [Source:HGNC Symbol;Acc:HGNC:4646] | 1936 | 0.368 | 0.0447 | No |
| 8 | PIH1D1 | PIH1 domain containing 1 [Source:HGNC Symbol;Acc:HGNC:26075] | 2667 | 0.283 | 0.0043 | No |
| 9 | H2AZ1 | H2A.Z variant histone 1 [Source:HGNC Symbol;Acc:HGNC:4741] | 2756 | 0.275 | 0.0134 | No |
| 10 | RUVBL2 | RuvB like AAA ATPase 2 [Source:HGNC Symbol;Acc:HGNC:10475] | 2778 | 0.272 | 0.0275 | No |
| 11 | AGO1 | argonaute RISC component 1 [Source:HGNC Symbol;Acc:HGNC:3262] | 3430 | 0.208 | -0.0110 | No |
| 12 | TAF1B | "TATA-box binding protein associated factor, RNA polymerase I subunit B [Source:HGNC Symbol;Acc:HGNC:11533]" | 3496 | 0.203 | -0.0044 | No |
| 13 | TBPL1 | TATA-box binding protein like 1 [Source:HGNC Symbol;Acc:HGNC:11589] | 3794 | 0.178 | -0.0172 | No |
| 14 | RELA | "RELA proto-oncogene, NF-kB subunit [Source:HGNC Symbol;Acc:HGNC:9955]" | 3942 | 0.161 | -0.0193 | No |
| 15 | DRAP1 | DR1 associated protein 1 [Source:HGNC Symbol;Acc:HGNC:3019] | 4462 | 0.116 | -0.0530 | No |
| 16 | STAT1 | signal transducer and activator of transcription 1 [Source:HGNC Symbol;Acc:HGNC:11362] | 4495 | 0.113 | -0.0489 | No |
| 17 | TP53 | tumor protein p53 [Source:HGNC Symbol;Acc:HGNC:11998] | 5511 | 0.032 | -0.1260 | No |
| 18 | MACROH2A1 | macroH2A.1 histone [Source:HGNC Symbol;Acc:HGNC:4740] | 5953 | -0.002 | -0.1602 | No |
| 19 | DR1 | down-regulator of transcription 1 [Source:HGNC Symbol;Acc:HGNC:3017] | 6369 | -0.033 | -0.1906 | No |
| 20 | EZH2 | enhancer of zeste 2 polycomb repressive complex 2 subunit [Source:HGNC Symbol;Acc:HGNC:3527] | 6562 | -0.047 | -0.2028 | No |
| 21 | H3-3B | H3.3 histone B [Source:HGNC Symbol;Acc:HGNC:4765] | 7351 | -0.106 | -0.2580 | No |
| 22 | HDAC1 | histone deacetylase 1 [Source:HGNC Symbol;Acc:HGNC:4852] | 7911 | -0.148 | -0.2929 | No |
| 23 | TAF4 | TATA-box binding protein associated factor 4 [Source:HGNC Symbol;Acc:HGNC:11537] | 7966 | -0.153 | -0.2882 | No |
| 24 | MMP12 | matrix metallopeptidase 12 [Source:HGNC Symbol;Acc:HGNC:7158] | 9285 | -0.254 | -0.3760 | No |
| 25 | H3-3A | H3.3 histone A [Source:HGNC Symbol;Acc:HGNC:4764] | 9503 | -0.274 | -0.3770 | No |
| 26 | SMARCA4 | "SWI/SNF related, matrix associated, actin dependent regulator of chromatin, subfamily a, member 4 [Source:HGNC Symbol;Acc:HGNC:11100]" | 10009 | -0.322 | -0.3976 | No |
| 27 | SMARCB1 | "SWI/SNF related, matrix associated, actin dependent regulator of chromatin, subfamily b, member 1 [Source:HGNC Symbol;Acc:HGNC:11103]" | 10117 | -0.333 | -0.3866 | No |
| 28 | KLF10 | Kruppel like factor 10 [Source:HGNC Symbol;Acc:HGNC:11810] | 10631 | -0.386 | -0.4042 | Yes |
| 29 | FOS | "Fos proto-oncogene, AP-1 transcription factor subunit [Source:HGNC Symbol;Acc:HGNC:3796]" | 10640 | -0.387 | -0.3824 | Yes |
| 30 | ZBTB17 | zinc finger and BTB domain containing 17 [Source:HGNC Symbol;Acc:HGNC:12936] | 10996 | -0.429 | -0.3852 | Yes |
| 31 | TBP | TATA-box binding protein [Source:HGNC Symbol;Acc:HGNC:11588] | 11138 | -0.448 | -0.3702 | Yes |
| 32 | AGO2 | argonaute RISC catalytic component 2 [Source:HGNC Symbol;Acc:HGNC:3263] | 11246 | -0.463 | -0.3517 | Yes |
| 33 | POU2F1 | POU class 2 homeobox 1 [Source:HGNC Symbol;Acc:HGNC:9212] | 11714 | -0.535 | -0.3570 | Yes |
| 34 | RRN3 | "RRN3 homolog, RNA polymerase I transcription factor [Source:HGNC Symbol;Acc:HGNC:30346]" | 11762 | -0.543 | -0.3293 | Yes |
| 35 | UBTF | upstream binding transcription factor [Source:HGNC Symbol;Acc:HGNC:12511] | 12261 | -0.665 | -0.3295 | Yes |
| 36 | REST | RE1 silencing transcription factor [Source:HGNC Symbol;Acc:HGNC:9966] | 12382 | -0.703 | -0.2982 | Yes |
| 37 | NPM1 | nucleophosmin 1 [Source:HGNC Symbol;Acc:HGNC:7910] | 12530 | -0.762 | -0.2655 | Yes |
| 38 | TAF1C | "TATA-box binding protein associated factor, RNA polymerase I subunit C [Source:HGNC Symbol;Acc:HGNC:11534]" | 12552 | -0.775 | -0.2223 | Yes |
| 39 | MYC | "MYC proto-oncogene, bHLH transcription factor [Source:HGNC Symbol;Acc:HGNC:7553]" | 12801 | -0.970 | -0.1854 | Yes |
| 40 | CEBPB | CCAAT enhancer binding protein beta [Source:HGNC Symbol;Acc:HGNC:1834] | 12804 | -0.973 | -0.1292 | Yes |
| 41 | BAZ2A | bromodomain adjacent to zinc finger domain 2A [Source:HGNC Symbol;Acc:HGNC:962] | 12879 | -1.166 | -0.0675 | Yes |
| 42 | TAF1 | TATA-box binding protein associated factor 1 [Source:HGNC Symbol;Acc:HGNC:11535] | 12881 | -1.192 | 0.0015 | Yes |
Table: GSEA details [plain text format]

  

Fig 2: GOMF\_CORE\_PROMOTER\_SEQUENCE\_SPECIFIC\_DNA\_BINDING      
 Blue-Pink O' Gram in the Space of the Analyzed GeneSet

  

Fig 3: GOMF\_CORE\_PROMOTER\_SEQUENCE\_SPECIFIC\_DNA\_BINDING: Random ES distribution      
 Gene set null distribution of ES for **GOMF\_CORE\_PROMOTER\_SEQUENCE\_SPECIFIC\_DNA\_BINDING**
